# Nature vs. Nurture: Disentangling the Influence of Inheritance, Incubation Temperature, and Post-Natal Care on Offspring Heart Rate and Metabolism in Zebra Finches

**DOI:** 10.3389/fphys.2022.892154

**Published:** 2022-05-10

**Authors:** Sydney F. Hope, Louise Schmitt, Olivier Lourdais, Frédéric Angelier

**Affiliations:** Centre d’Etudes Biologiques de Chizé, CNRS—La Rochelle Université, Villiers en Bois, France

**Keywords:** incubation temperature, heritability, metabolic rate, thermoregulation, embryonic heart rate, cost of thermoregulation

## Abstract

A historic debate in biology is the question of nature vs. nurture. Although it is now known that most traits are a product of both heredity (“nature”) and the environment (“nurture”), these two driving forces of trait development are rarely examined together. In birds, one important aspect of the early developmental environment is egg incubation temperature. Small changes (<1°C) in incubation temperature can have large effects on a wide-array of offspring traits. One important trait is metabolism, because it is related to life-history traits and strategies, organismal performance, and energetic and behavioral strategies. Although it has been shown that embryonic and post-hatch metabolism are related to egg incubation temperature, little is known about how this may vary as a function of genetic differences or post-hatching environmental conditions. Here, we investigated this question in zebra finches (*Taeniopygia guttata*). We experimentally incubated eggs at two different temperatures: 37.5°C (control), which is optimal for this species and 36.3°C (low), which is suboptimal. We first measured embryonic heart rate as a proxy of embryonic metabolic rate. Then, at hatch, we cross-fostered nestlings to differentiate genetic and pre-hatching factors from post-hatching environmental conditions. When offspring were 30 days-old, we measured their resting metabolic rate (RMR; within the thermoneutral zone) and thermoregulatory metabolic rate (TMR; 12°C; birds must actively thermoregulate). We also measured RMR and TMR of all genetic and foster parents. We found that embryonic heart rate was greater in eggs incubated at the control temperature than those at the low temperature. Further, embryonic heart rate was positively related to genetic father RMR, suggesting that it is both heritable and affected by the pre-natal environment. In addition, we found that post-hatch metabolic rates were positively related to genetic parent metabolic rate, and interactively related to incubation temperature and foster mother metabolic rate. Altogether, this suggests that metabolism and the energetic cost of thermoregulation can be influenced by genetics, the pre-natal environment, and the post-natal environment. Our study sheds light on how environmental changes and parental care may affect avian physiology, as well as which traits may be susceptible to natural selection.

## 1 Introduction

Whole-organism metabolism is a fundamental aspect animal physiology, and thus understanding the drivers of individual variation in metabolism is crucial (Burton et al., 2011; White and Kearney, 2013; Pettersen et al., 2018). Resting metabolic rate (RMR) represents an individual’s minimum energy requirements for self-maintenance (Daan et al., 1990; Bryant, 1997), excluding physiological processes such as thermoregulation, digestion, and activity (McNab, 1997). RMR is important for understanding basal metabolic rate, and is also related to individual life history traits and strategies, performance, energetic strategies, behavior, reproductive success, and survival (Careau et al., 2008; Biro and Stamps, 2010; Williams et al., 2010; Careau and Garland, 2012; Rønning et al., 2016; Auer et al., 2018; Pettersen et al., 2018). While metabolic rate varies depending on current environmental conditions (Broggi et al., 2004; Norin and Metcalfe, 2019), there is evidence that, across taxa, individual differences in metabolic rate are repeatable (Nespolo and Franco, 2007; Careau et al., 2008; Broggi et al., 2009; Réveillon et al., 2019; Baškiera and Gvoždík, 2021; Dezetter et al., 2021) and heritable (reviewed in Pettersen et al., 2018). Further, conditions during early development and parental effects can also have lasting effects on individual metabolism (Burton et al., 2011). For example, in oviparous species, maternal hormone deposition to eggs can affect offspring post-hatch metabolic rate (Groothuis et al., 2005; Tobler et al., 2007; Nilsson et al., 2011). However, little is known about how different drivers (e.g., heritability and parental effects) may interact to influence metabolism (Burton et al., 2011; White and Kearney, 2013; Pettersen et al., 2018; McFarlane et al., 2021). Understanding the sources of inter-individual variation in metabolism will shed light on how environmental changes, parental care decisions, and natural selection can shape this important aspect of physiology.

In birds, some of the most important sources of variation in offspring physiology arise from parental care decisions. Aside from important maternal effects during egg-laying (e.g., nutrient/hormone transfer to eggs; (Groothuis et al., 2005; Tobler et al., 2007; Nilsson et al., 2011), two essential ways in which parents must ensure proper offspring development are through egg incubation and post-hatch nestling care. Incubation is necessary for eggs to hatch (Deeming and Ferguson, 1991), but energetically costly and time consuming for parents (Tinbergen and Williams, 2002; Nord and Williams, 2015). In turn, incubation investment varies among parents, due to factors such as ambient temperature, clutch size, parental experience, and individual quality (Aldrich and Raveling, 1983; Haftorn and Reinertsen, 1985; Conway and Martin, 2000; Ardia and Clotfelter, 2007; Coe et al., 2015; Amininasab et al., 2016; Hope et al., 2020; Williams et al., 2021). This causes incubation temperatures to vary both among and within nests (Boulton and Cassey, 2012; Coe et al., 2015; Hope et al., 2021). Importantly, small differences in temperature can have large effects on offspring physiology, such as metabolic rate, thermoregulation, glucocorticoid hormone levels, immune function, and telomere length (Nord and Nilsson, 2011; DuRant et al., 2013; Hepp et al., 2015; Wada et al., 2015; Stier et al., 2020; Hope et al., 2021). Similarly, in altricial species, parental food provisioning is essential for the proper growth and development of offspring. However, parents vary in their nestling provisioning rates due to factors such as food availability, the sex of the parent, parental experience, ambient temperature, brood size, and predation risk (Wright et al., 1998; Wiebe and Neufeld, 2003; Barba et al., 2009; Low et al., 2012; Ghalambor et al., 2013), with some evidence that provisioning behavior is repeatable and that some individuals are consistently “good” parents (Schwagmeyer and Mock, 2003). As with incubation temperature, differences in provisioning can affect offspring morphology and physiology. For example, food limitation during nestling development is related to low body masses, slow growth rates, altered glucocorticoid hormone levels, higher metabolic rates, and lower survival (Lepczyk and Karasov, 2000; Killpack and Karasov, 2012; Schmidt et al., 2012).

There is evidence that genetics, incubation temperature, and post-hatch parental care can influence both juvenile and adult avian metabolic rate. For example, avian RMR has been shown to be repeatable within individuals and heritable through adulthood (Bech et al., 1999; Rønning et al., 2005, 2007; Broggi et al., 2009; Nilsson et al., 2009). Further, studies have found that eggs incubated at lower temperatures have slower embryonic development and lower embryonic metabolic rates (DuRant et al., 2011; Stier et al., 2020) but, after hatching, produce offspring that have higher RMR early in life compared to those incubated at a warmer temperature (Nord and Nilsson, 2011; Wada et al., 2015). Moreover, environmental stressors during the post-hatch development, such as glucocorticoid exposure (Spencer and Verhulst, 2008; Dupont et al., 2019), food restriction (Moe et al., 2005; Criscuolo et al., 2008; Rønning et al., 2009; Careau et al., 2014), and sibling competition (Burness et al., 2000; Verhulst et al., 2006), can have long-lasting effects on offspring RMR. Additionally, another important aspect of metabolism is thermoregulatory metabolic rate (TMR), which is the metabolic rate organisms express under challenging thermal conditions, and represents the metabolic cost associated with thermoregulation (Broggi et al., 2004; Carleton and Rio, 2005; Nzama et al., 2010; DuRant et al., 2012; Dupont et al., 2019). Although less-often studied compared to RMR, there is also some evidence that avian TMR can be affected by incubation temperature and the post-hatch environment. For example, one study found that wood ducks (*Aix sponsa*) incubated at a lower temperature had higher TMR than those incubated at a warmer temperature (DuRant et al., 2012). Further, one study found that house sparrows (*Passer domesticus*) with increased glucocorticoid exposure during post-hatch development had lower TMR than control nestlings (Dupont et al., 2019). However, despite the evidence for the influence of genetics and pre- and post-hatch parental effects on both avian RMR and TMR, no study to date has investigated whether these different drivers may interact to affect metabolism.

In this study, we investigated whether genetics, incubation temperature, and/or post-hatch parental care interact to explain individual variation in avian RMR or TMR. To do this, we incubated zebra finch (*T. guttata*) eggs at two different temperatures: 37.5°C (control), which is optimal for this species and 36.3°C (low), which is suboptimal in this species, as shown in other studies (Wada et al., 2015; Berntsen and Bech, 2016). During incubation, as a proxy of embryonic metabolic rate, we measured embryonic heart rate (Sheldon et al., 2018). Then, at hatch, we cross-fostered nestlings to decouple genetic and pre-hatching factors from post-hatching environmental conditions. Lastly, we measured the RMR and TMR of all offspring at Day 30 (i.e., nutritional independence), and of all parents after reproduction had ended. Our main hypothesis was that offspring metabolism is shaped through a combination of inheritance, incubation conditions, and post-natal care. We tested the following predictions:

1) Embryonic heart rate and offspring metabolic rate on Day 30 are positively related to the metabolic rate of genetic parents (i.e., metabolic rate is heritable; Rønning et al., 2007; Nilsson et al., 2009).
2) Lower incubation temperatures lead to slower embryonic heart rates (Rubin, 2019; Stier et al., 2020), but higher RMR (Nord and Nilsson, 2011; Wada et al., 2015) and TMR (DuRant et al., 2012) at Day 30.
3) We considered the relationship between foster parent and offspring metabolic rate to be representative of the overall influence of the post-hatch environment and predicted that offspring metabolic rate would also be related to the metabolic rate of foster parents.

Along with these predictions, we also tested for interactive effects among our incubation temperature treatment and parental metabolism, with the expectation that offspring metabolic rate and embryonic heart rate may have different relationships with parental metabolic rate, depending on the incubation temperature treatment.

## 2 Materials and Methods

### 2.1 General Husbandry and Breeding

We used a breeding colony of zebra finches (*T. guttata*; *N* = 20 pairs; “parents”) housed at the CEBC (CNRS) for this study. We first housed the 40 birds together in an indoor aviary for 10 days and we formed pairs based on mating behaviors that we observed (e.g., singing, proximity, etc.). We then housed pairs in cages (47.5 × 38 × 51 cm) with external nest boxes (12 × 13 × 16 cm). Ambient temperature was kept at a constant 22°C and the photoperiod was set to a 14:10 day:night cycle, for all aspects of the study, including pair formation, reproduction, and nestling rearing. We provided birds with ∼10 g of alfalfa hay every day, and then ∼1 g of coconut fiber once the hay completely covered the bottom of the nest box. We misted pairs with water once per day until their first egg was laid, to stimulate reproduction. We provided birds with *ad libitum* food (Versele-Laga Prestige Tropical Finches seed mix), water supplemented with vitamins, cuttlefish bone, and grit. We also gave birds ∼2 g of chopped hard-boiled eggs (including the shells) every day from pair formation until nestling Day 30, along with endives and millet sprays once per week (Olson et al., 2014). All procedures in this study were approved by the national ethics committee for animal experimentation under file number APAFIS#23727-2020011311559318.

### 2.2 Egg Incubation

We checked nest boxes daily at 10:00. Once an egg was found, we marked it with a unique ID using a small marker, weighed it, and placed it in an incubator (Brinsea^©^ Ovation 28 Advance digital egg incubator) at one of two temperatures. We followed an incubation protocol similar to Wada et al. (2015). The “control” incubator was set at a constant 37.5°C (± 0.1 [SD]), which is likely optimal for zebra finches. The “low” incubator was set at a constant 36.3°C (± 0.1 [SD]), which is within the natural range of zebra finch incubation temperatures, but there is evidence that it produces suboptimal offspring phenotypes in this species (Wada et al., 2015). Both incubators were set at a humidity of 55%. We verified the temperature and humidity by placing iButton^©^ (Hygrochron DS 1923, Maxim Integrated^™^) temperature loggers inside of each incubator. We randomly assigned the incubation treatment to the first laid egg of each breeding pair, and then systematically alternated among temperature treatments for each subsequent egg for the entire length experiment. Multiple clutches from each breeding pair were used in this study, to attain a sufficient sample size. During artificial incubation, we gave parents fake clay eggs to incubate so that they stayed in the breeding phase. One day before the predicted hatching date (day 13 for “control” eggs and day 14 for “low” eggs), we transferred eggs to a hatcher that was set at a temperature of 37.5°C and 67% humidity.

### 2.3 Embryonic Heart Rate

Embryonic heart rate is correlated with embryonic oxygen consumption (Du et al., 2010), and thus can be used as a proxy for energy expenditure during embryonic development. We measured embryonic heart rate by placing eggs in the Buddy digital egg monitor (Vetronic Services, Abbotskerswell, Devon, United Kingdom). We considered that a reading was reliable when the curve and heart rate outputs were relatively consistent for ∼10 s (Sheldon et al., 2018). At each timepoint (see below), we took three repeated heart rate measures within 3 min of taking each egg (individually) out of the incubator and noted the time (seconds) that it took to take each measure. If any/all of the readings were unreliable (e.g., due to embryo movement; Sheldon et al., 2018), they were excluded from the analyses. All readings were taken in a room at a constant temperature of 22°C. If there was a consistent heart rate reading of “0”, we candled eggs and determined if they were infertile or had died during development.

We measured heart rates of embryos after 11, 12 and 13 days for incubation for “control” eggs and after 12, 13 and 14 days of incubation for “low” eggs. These three measures are hereafter referred to as “readings 1, 2 and 3”. We chose these days because the incubation period of “control” eggs is about 1 day shorter than that of and “low” eggs (Table 1) and, thus, we chose to investigate differences in heart rate among embryos at the same stage of development (i.e., “developmental age”), instead of after the same number of days (i.e., “calendar age”). We validated that our embryonic heart rate results were not driven by differences in eggshell temperature, and that they were not affected by our choice of using “developmental age” instead of “calendar age” (see Supplementary Appendix S1). We placed eggs in the hatcher after the final heart rate readings.

**TABLE 1:** Summary statistics of hatch success, incubation period, body mass, and metabolic rate.

Variable	Incubation temperature			
	Control (37.5°C)		Low (36.3°C)	
	Mean ± SE	N	Mean ± SE	N
Hatch success^a^	25.9%	135	23.9%	134
Incubation period (days)	13.8 ± 0.08	35	15.1 ± 0.06	32
Body mass day 0 (g)^b^	0.80 ± 0.02	33	0.83 ± 0.02	32
Body mass day 30 (g)	14.6 ± 0.39	20	14.4 ± 0.36	15
TMR (VO_2_)	6.88 ± 0.16	20	6.93 ± 0.25	15
RMR (VO_2_)	3.18 ± 0.08	20	3.06 ± 0.09	15

a Excludes infertile and cracked eggs.

b Excludes two individuals that hatched but died in the incubator.

### 2.4 Nestling Monitoring

We checked the hatcher for hatching multiple times each day and, at a minimum, once at 9:00 and once at 17:00. Once hatched, we weighed and marked nestlings by removing distinct patches of down feathers (Adam et al., 2014). Then, we cross-fostered nestlings. We gave parents up to two nestlings, which were never from the same incubation treatment, and nestlings were never more than 1 day apart in age. We housed nestlings with their foster parents until independence (i.e., Day 30), and then we conducted the metabolism measurements. Afterward, we housed independent offspring in sex-specific communal cages for use in future studies. We banded nestlings on Day 10 and determined their sex using plumage characteristics on Day 30.

### 2.5 Metabolism

We quantified energy metabolism in both parents and offspring by measuring oxygen consumption rates using multichannel open-circuit respirometry (Sable Systems Int., Las Vegas, NV, United States; Brischoux et al., 2017). We measured offspring when they were 32 ± 2.9 (range: 28–39) days-old, and we measured parents after they had finished reproduction [75 ± 26 days after their last foster nestling reached Day 30 (for those that successfully raised at least one nestling); 102 ± 20 days since their last laid egg (all birds)]. We measured metabolism of each bird twice: once at 32°C (RMR; within the thermoneutral zone; Calder, 1964) and once at 12°C (TMR; when birds must actively thermoregulate; Dupont et al., 2019), to determine whether incubation temperature might affect energy expenditure during a thermal challenge. Measurements at different temperatures were conducted on consecutive days and the order was randomized among incubation temperature treatments. We weighed birds at ∼20:15 and began respirometry at ∼20:30. Up to 7 birds were measured each night, and the system measured oxygen consumption of each bird for 10 min and systematically alternated among chambers, with 15 min of baseline reading (empty chamber) each time a full cycle was completed. Birds were removed and weighed again at ∼8:30 the next morning. We did not analyze the first 3 h of data because this was the time when birds fasted (Wada et al., 2015). Oxygen flow was set at ∼350 ml/min, and O_2_ at 20.95%, which was recalibrated each night.

To calculate metabolic rate, we first chose the value of oxygen consumption for each 10 min run of each individual that was the lowest and most consistent, using the computer software ExpeData (Sable Systems). Then, we calculated metabolic rate using the Hoffman Equation for VO_2_ (ml/h), and then corrected for body mass (ml/h/g). Lastly, we calculated the mean VO_2_ of all runs of each individual to obtain the final VO_2_ value (ml/h/g) for each individual.

### 2.6 Statistical Analyses

We conducted all statistical analyses using R v 3.5.1 (R Core Team, 2018). We reduced models using stepwise backwards elimination of non-significant terms (*p* > 0.10), starting with non-significant interactions. After eliminating the term with the highest *p*-value, we reran the model and continued this process until only significant (*p* < 0.05) or marginally significant (0.05 < *p* < 0.10) terms remained in the model. Incubation temperature was always treated as a categorical variable. We ensured that all models met the assumptions of normal and homoscedastic residuals by investigating histograms of residuals, normal quantile plots, and fitted vs. residuals plots. We verified that models met the assumption of non-multicollinearity by investigating the variance inflation factors (*vif*). Further, all continuous independent variables that were used in interactions were scaled and centered to reduce multicollinearity. We used the package *lme4* (Bates et al., 2015) for mixed effects models and *emmeans* (Lenth, 2018) for post-hoc tests, including slope comparisons for interactions. *p*-values were calculated using the *Anova* function using the *car* (Fox and Weisberg, 2011) package. *R* ^*2*^ values for mixed effects models were calculated using the *MuMln* package (Bartoń, 2018). Figures were created using the *plyr* (Wickham, 2011) and *ggplot2* (Wickham, 2016) packages. Two male parents died for reasons unrelated to the experiment before parental metabolic rates were measured, and thus their RMR, TMR, and ΔMR were not able to be included in the analyses. Neither of these males was a genetic father to any offspring that lived until Day 30 in this study, and only one of these males was a foster father to a single individual that lived until Day 30.

First, to determine whether embryonic heart rate was related to incubation temperature and/or parent metabolism, we built one linear mixed effect model with heart rate as the dependent variable. The independent variables were incubation temperature, reading (1, 2 or 3), the time it took to take the measurement (seconds), the RMR of both the genetic mother and father, along with all two-way interactions with incubation temperature. Parent TMR was not included in this model because 1) we predicted only that parental RMR would be related with embryonic metabolism, measured when embryos were at warm temperatures (i.e., incubation) and 2) parent TMR and RMR were correlated (*r* = 0.42; *p* < 0.01), and thus including them both in the model would increase multicollinearity. We also included whether the egg hatched or not as an independent variable, and egg mass as a covariate. Egg ID was included as a random effect to control for repeated measures.

Second, to determine whether offspring metabolism was related to incubation temperature and the temperature at which the measurement was taken, we built one linear mixed effects model. Offspring metabolism (VO_2,_ ml/h/g) was the dependent variable, and it was log-transformed to meet model assumptions. The independent variables were incubation temperature, the temperature of the measurement (12°C or 32°C), and their interaction. Sex and age were also included as covariates, as well as the interaction between incubation temperature and sex. Individual ID, genetic parent ID, and foster parent ID were included as random effects to account for repeated measures within individuals and among siblings.

Next, we determined whether offspring metabolism was related to parental metabolism. First, we calculated heritability (*h* ^*2*^) as the slope of the regression between the mean value of genetic parent metabolism (either RMR or TMR) and offspring metabolism (Åkesson et al., 2008; Wray and Visscher, 2008). Then, to examine relationships among all parents (genetic and foster; separated by sex), and to test whether there was an interactive effect of incubation temperature and parental metabolism, we built three linear models. For all models, the dependent variable was offspring metabolism (VO_2,_ ml/h/g) and the independent variables were incubation temperature, the metabolism of the genetic mother, genetic father, foster mother, and foster father, along with all two-way interactions with incubation temperature. The difference among the three models was that the first included only RMR data (both parents and offspring), the second included only TMR data, and the third used the difference between TMR and RMR (i.e., additional amount of energy expended during thermoregulation; hereafter ΔMR) for all individuals.

Lastly, to determine whether embryonic heart rate and offspring metabolism (at Day 30) were correlated within individuals, we built one linear mixed effect model. The dependent variable was embryonic heart rate, and only individuals that lived until the metabolic measurement (∼Day 30) were included in the model. The independent variables were incubation temperature, reading (1, 2 or 3), offspring RMR, and all two-way interactions with incubation temperature. Offspring TMR was not included in this model because 1) we predicted only that offspring RMR would be related with embryonic metabolism, measured when embryos were at warm temperatures (i.e., incubation) and 2) offspring TMR and RMR were correlated (*r* = 0.73; *p* < 0.001), and thus including them both in the model would increase multicollinearity. We also included sex and its interaction with incubation temperature in this analysis because, contrary to the first analysis, we had data on the sex of all individuals (i.e., only individuals that lived until Day 30 were included). The time it took to take the measurement (seconds) and egg mass were also included as covariates. Egg ID, genetic parent ID, and foster parent ID were included as random effects to control for repeated measures among siblings.

## 3 Results

### 3.1 Hatching Success, Incubation Period, and Body Mass

Summary statistics for hatching success, incubation period, and nestling body mass (Days 0 and 30) are reported in Table 1. There were no differences in hatching success, body mass at Day 0, or body mass at Day 30 between incubation temperature treatment groups (all *p* > 0.25; simple linear models). However, eggs incubated at the lower temperature had a longer incubation period than those incubated at the control temperature (*p* < 0.001).

### 3.2 Embryonic Heart Rate: Relationship With Incubation Temperature and Parental Metabolism

**FIGURE 1:**
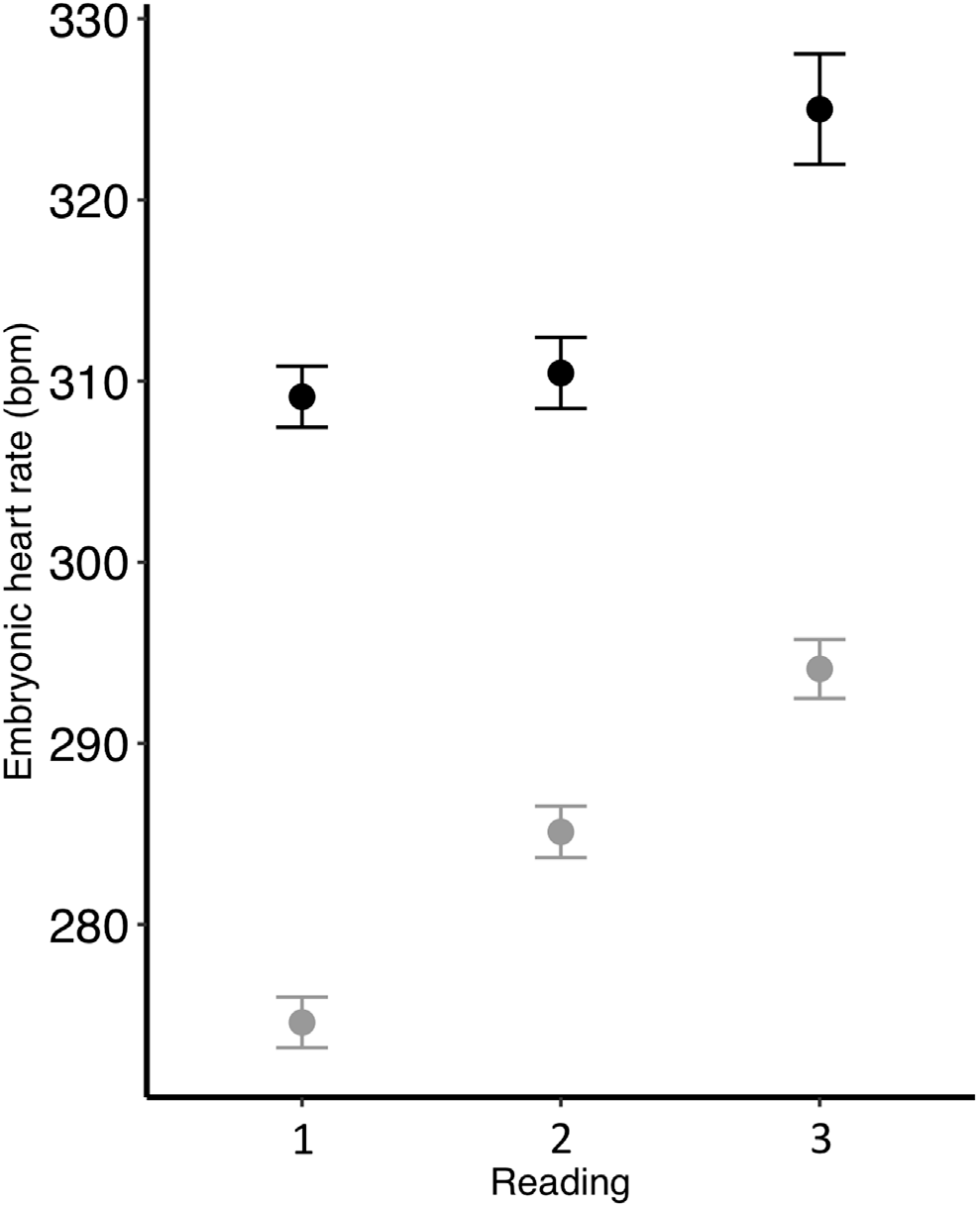
Zebra finch embryonic heart rate (beats per minute; bpm) at three different time points during development and incubated at two different temperatures (black = control; gray = low). To correct for different developmental rates, readings were taken on control eggs after (1) 11, (2) 12, and (3) 13 days of incubation, while readings were taken on low eggs after (1) 12, (2) 13, and (3) 14 days. Mean ± SE are shown.

**FIGURE 2:**
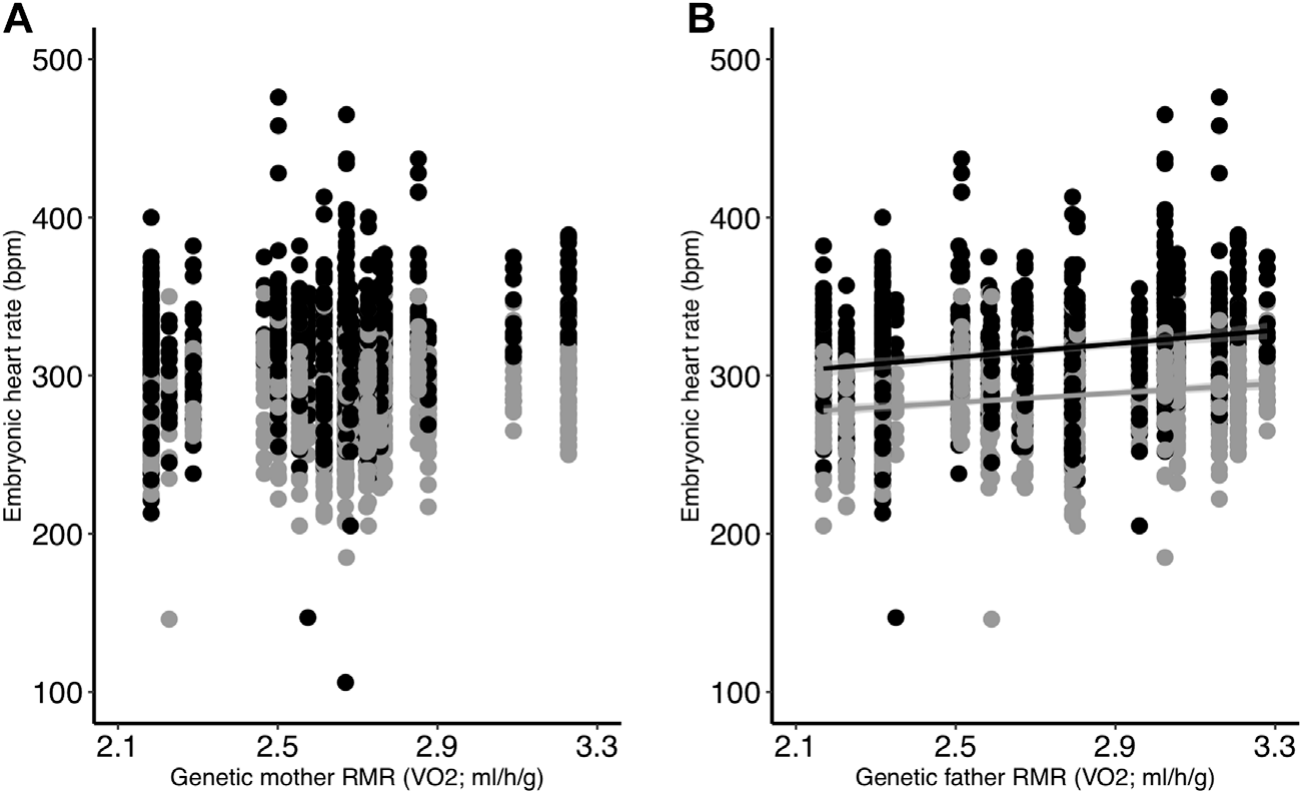
The relationships between egg heart rate (bpm) and **(A)** genetic mother RMR and **(B)** genetic father RMR. Eggs were incubated at two different temperatures (black = control; gray = low). All egg heart rate measurements are shown. Regression lines are included only for significant relationships.

Embryonic heart rate was related to both incubation temperature and parent metabolic rate. We found that heart rate was greater in embryos from the control treatment compared to the low treatment (*p* < 0.001; Table 2; Figure 1), and that heart rate increased throughout the course of incubation (reading: *p* < 0.001; Table 2). There was an interactive effect of incubation temperature and reading on heart rate (*p* < 0.001; Table 2), and post-hoc tests revealed significant differences for all pairwise comparisons (all *p* < 0.001), except between the heart rate of control embryos on days 11 and 12 (*p* > 0.99; Figure 1). Although embryonic heart rate was significantly related to mother RMR in the full model, it was not retained in the final model (Table 2; Figure 2A). However, embryonic heart rate was positively correlated with father RMR (*p* = 0.0003; Table 2; Figure 2B). There was also a relationship between whether or not the egg hatched and its heart rate (*p* = 0.049; Table 2), where embryos with greater heart rates were more likely to hatch. Further, heart rate increased as egg mass increased (*p* < 0.001; Table 2), and heart rate decreased with the time that it took to take the measurement (*p* < 0.001; Table 2).

**TABLE 2:** Full and reduced models investigating the relationship of embryonic heart rate with incubation temperature and parental metabolism.

	Embryonic heart rate (bpm)^a^	
	N_control_ = 107 eggs; 839 readings	
	N_low_ = 117 eggs; 997 readings	
	Full model	
	R^2^m = 0.43; R^2^c = 0.70	
Term	F	*p*
Incubation temperature	**120.88**	**<0.0001**
Reading (1, 2, or 3)	**26.41**	**<0.0001**
Time until measurement (seconds)	**425.50**	**<0.0001**
Egg mass	**19.49**	**<0.0001**
Hatched (yes/no)	**4.18**	**0.042**
Mother RMR	**4.44**	**0.036**
Father RMR	0.86	0.36
Incubation X reading	**10.01**	**<0.0001**
Incubation X time	2.49	0.12
Incubation X Mother RMR	1.77	0.18
Incubation X Father RMR	0.42	0.52
	**Reduced model**	
		**R^2^m = 0.43; R^2^c = 0.70**	
Incubation temperature	**140.31**	**<0.0001**
Reading (1, 2, or 3)	**27.47**	**<0.0001**
Time until measurement (seconds)	**766.86**	**<0.0001**
Egg mass	**19.70**	**<0.0001**
Hatched (yes/no)	**3.91**	**0.049**
Father RMR	**13.55**	**0.0003**
Incubation X Reading	**9.48**	**<0.0001**

Bold values indicate statistical significance.

a Egg ID was included as a random effect to control for repeated measures.

### 3.3 Offspring Metabolic Rate: Relationship With Incubation Temperature and Parental Metabolism

**FIGURE 3:**
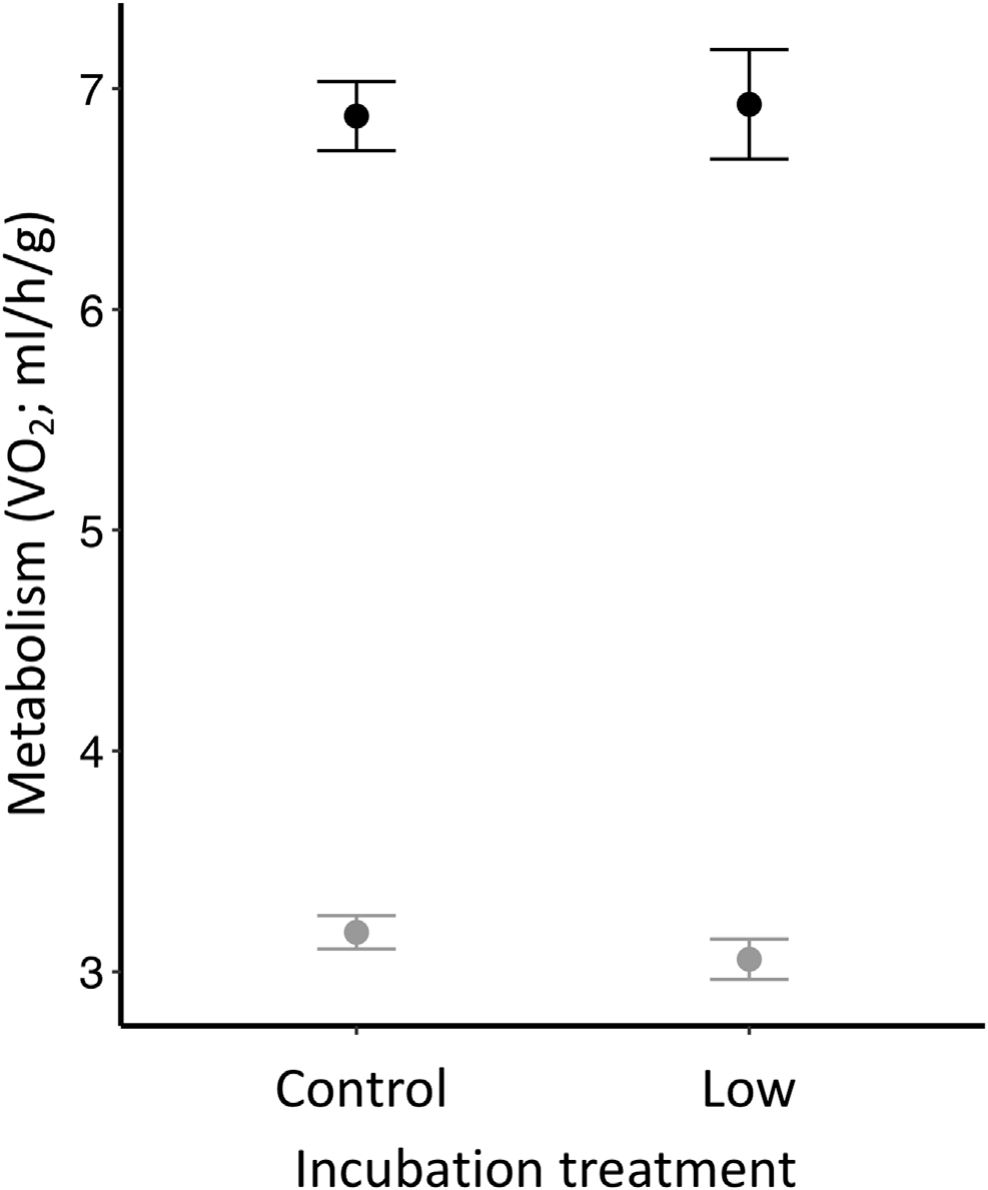
Zebra finch metabolic rate (VO_2_; ml/h/g) measured at two different temperatures (black = TMR: 12°C; gray = RMR: 32°C). Individuals were measured at Day 30 and had hatched from eggs incubated at two different temperatures (i.e., control or low).

Although we found no differences in metabolic rate among offspring incubated at different temperatures or between sexes (Table 3), we found that offspring metabolism was related to the temperature at which the measurement was taken. As expected, metabolism was greater when the measurement was taken at 12°C (TMR) than at 32°C (RMR) (*p* < 0.0001; Table 3; Figure 3).

**TABLE 3:** Full and reduced models investigating the effects of incubation temperature and temperature of measurement on offspring metabolism at Day 30.

	Offspring VO_2_ (ml/h/g)^a^ ^,^ ^b^	
	N_control_ = 20; N_low_ = 15	
	Full model	
	R^2^m = 0.93; R^2^c = 0.98	
Term	F	*p*
Incubation temperature	0.98	0.33
Temperature (12 or 32 °C)	**1847.1**	**<0.0001**
Sex	3.30	0.08^*^
Age	0.08	0.78
Incubation X Temperature	2.54	0.12
Incubation X Sex	1.55	0.22
	**Reduced model**	
	**R^2^m = 0.92; R^2^c = 0.98**	
Temperature (12 or 32 °C)	**3380.8**	**<0.0001**

Bold values indicate statistical significance and asterisks (*) indicate marginal significance.

a log-transformed to meet model assumptions.

b Parent (genetic and foster) and individual IDs were included as random effects in model.

**FIGURE 4:**
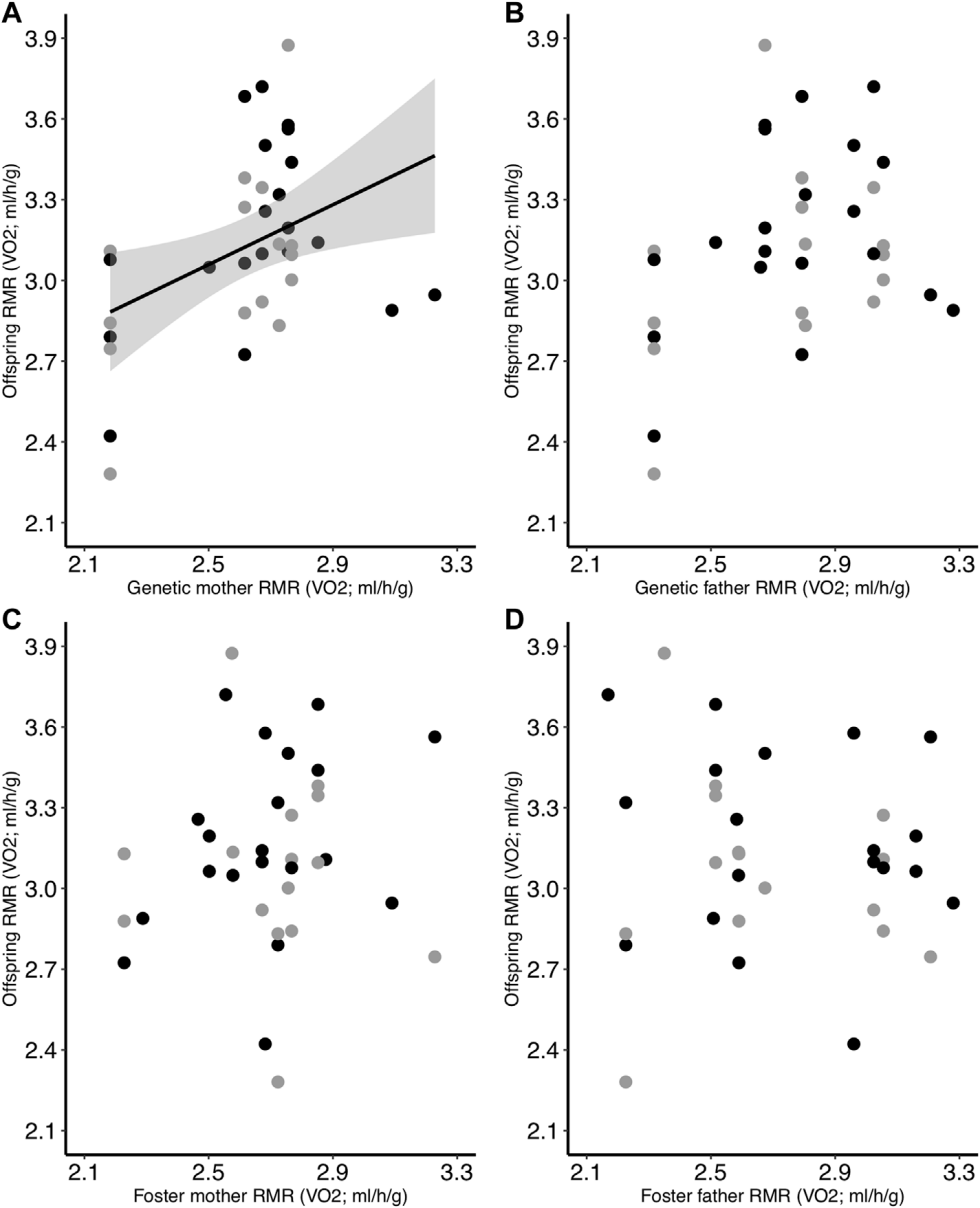
Relationship between offspring RMR on Day 30 and the RMR of the **(A)** genetic mother, **(B)** genetic father, **(C)** foster mother, and **(D)** foster father. Offspring had been incubated at two different temperatures as eggs (black = control; gray = low). Regression lines are included only for significant relationships.

The regression of mean genetic parent RMR with offspring RMR revealed that RMR was significantly heritable [*h* ^*2*^ = 0.53 ± 0.22 (SE), *p* = 0.02]. When we examined the relationships of all parents (genetic and foster) as individual factors, we found that genetic mother RMR was positively related to offspring RMR (*p* = 0.014; Table 4; Figure 4A). However, offspring RMR was not related to any other parental RMR, and there were no interactive relationships with incubation temperature (Table 4; Figure 4B–D).

**TABLE 4:** Full and reduced models investigating the relationship of offspring metabolism at Day 30 with interactions between incubation temperature and parental metabolism.

	Response variable: Offspring metabolism (ml/h/g)					
	N_control_ = 20;		N_control_ = 20;		N_control_ = 20;	
	N_low_ = 15		N_low_ = 15		N_low_ = 15	
	Includes RMR data		Includes TMR data		Includes difference between TMR and RMR (ΔMR)	
	Full models					
	Multiple R^2^ = 0.42; Adjusted R^2^ =0.20		Multiple R^2^ = 0.32; Adjusted R^2^ =0.06		Multiple R^2^ = 0.45; Adjusted R^2^ =0.25	
Term	F	p	F	p	F	p
Incubation temperature	0.006	0.94	0.079	0.78	0.89	0.35
Genetic mother metabolism	0.24	0.63	0.81	0.38	0.42	0.53
Foster mother metabolism	3.94	0.059*	0.034	0.85	0.50	0.49
Genetic father metabolism	0.11	0.74	0.57	0.46	0.58	0.45
Foster father metabolism	1.69	0.21	1.13	0.30	0.68	0.42
Incubation X Genetic mother	**4.31**	**0.049**	0.67	0.42	1.20	0.28
Incubation X Foster mother	0.87	0.36	3.92	0.059*	**4.42**	**0.046**
Incubation X Genetic father	2.82	0.11	0.14	0.72	1.35	0.26
Incubation X Foster father	3.32	0.081*	1.60	0.22	1.07	0.31

	**Reduced models**					
	**Multiple R^2^ = 0.17; Adjusted R^2^ =0.14**		**Multiple R^2^ = 0.25; Adjusted R^2^ =0.15**		**Multiple R^2^ = 0.38; Adjusted R^2^ =0.30**	
Incubation temperature	—	—	0.11	0.74	1.26	0.27
Genetic mother metabolism	**6.73**	**0.014**	—	—	—	—
Foster mother metabolism	—	—	0.017	0.90	1.10	0.30
Genetic father metabolism	—	—	3.01	0.093*	**4.20**	**0.049**
Incubation X Foster mother	—	—	**5.01**	**0.033**	**5.57**	**0.025**

Bold values indicate statistical significance and asterisks (*) indicate marginal significance.

The regression of mean genetic parent TMR with offspring TMR revealed that the heritability of TMR was not statistically significant [*h* ^*2*^ = 0.38 ± 0.28 (SE), *p* = 0.19]. However, when examining parents separately (i.e., all genetic and foster parents as separate independent variables), we found a trend that genetic father TMR was positively related to offspring TMR (*p* = 0.093; Table 4). Further, there was an interactive effect of incubation temperature and foster mother TMR on offspring TMR (*p* = 0.033; Table 4), where the TMR of offspring from the control group was not related to foster mother TMR (slope estimate: 0.022; confidence interval: −0.32 to 0.37) while the TMR of offspring from the low group was negatively related to the TMR of their foster mother (slope estimate: −0.56; confidence interval: −0.95 to −0.16). There were no relationships between offspring TMR and their genetic mother or foster father (Table 4).

**FIGURE 5:**
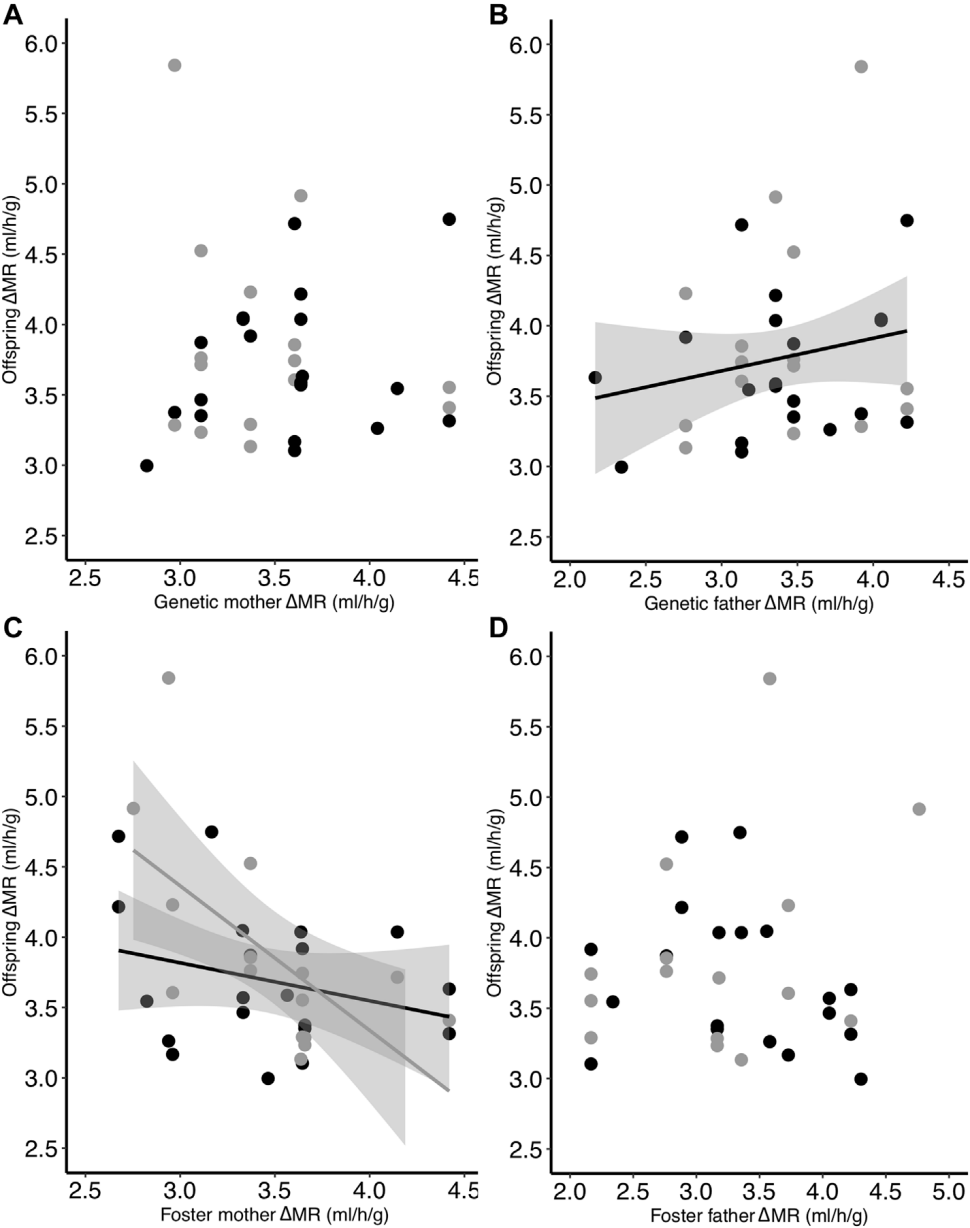
Relationship between the difference in offspring TMR and RMR (ΔMR) on Day 30 and the ΔMR of the **(A)** genetic mother, **(B)** genetic father, **(C)** foster mother, and **(D)** foster father. Offspring had been incubated at two different temperatures as eggs (black = control; gray = low). Regression lines are included only for significant relationships.

Similar to TMR, the heritability of ΔMR (i.e., TMR−RMR) was not statistically significant [*h* ^*2*^ = 0.18 ± 0.27 (SE), *p* = 0.51]. However, when examining parents separately, although there was no relationship between genetic mother ΔMR and offspring ΔMR (Table 4; Figure 5A), we found that there was a significant positive relationship between genetic father ΔMR and offspring ΔMR (*p* = 0.049; Table 4; Figure 5B). There was also an interactive effect of incubation temperature and foster mother ΔMR on offspring ΔMR (*p* = 0.025; Table 4; Figure 5C). This relationship mimicked that of TMR: the ΔMR of offspring from the control group was not related to foster mother ΔMR (slope estimate: −0.12; confidence interval: −0.34 to 0.11; Figure 5C) while the ΔMR of offspring from the low group was negatively related to the ΔMR of their foster mother (slope estimate: −0.55; confidence interval: −0.85 to −0.25; Figure 5C). There was no relationship between offspring ΔMR and that of their foster father (Table 4; Figure 5D).

### 3.4 Embryonic Heart Rate and Metabolic Rate: Relationship Within Individuals

Embryonic heart rate and offspring RMR were not correlated within individuals (Table 5). Further, there were no significant interactive effects of incubation temperature and RMR. The only terms that remained in the model (Table 5) were incubation temperature (*p* < 0.001), reading (*p* < 0.001), time to take the measurement (*p* < 0.001), and incubation temperature x reading (*p* < 0.001), as already found previously in Section 3.2.

**TABLE 5:** Full and reduced models investigating the relationship between embryonic heart rate and Day 30 metabolism.

	Embryonic heart rate (bpm)^a^	
	N_control_ = 20 eggs^b^;163 readings	
	N_low_ = 15 eggs^b^; 132 readings	
	Full model	
	R^2^m = 0.58; R^2^c = 0.76	
Term	F	p
Incubation temperature	1.95	0.18
Reading (1, 2, or 3)	**83.26**	**<0.0001**
Time until measurement (seconds)	**155.24**	**<0.0001**
Egg mass	3.05	0.11
Offspring RMR	0.95	0.34
Sex	**5.84**	**0.03**
Incubation X Reading	**10.52**	**<0.0001**
Incubation X Offspring RMR	3.02	0.09*
Incubation X Sex	2.33	0.15
	**Reduced model**	
	**R^2^m = 0.53; R^2^c = 0.76**	
Incubation temperature	**20.61**	**<0.0001**
Reading (1, 2, or 3)	**83.30**	**<0.0001**
Time until measurement (seconds)	**154.7**	**<0.0001**
Incubation X Reading	**10.60**	**<0.0001**

Bold values indicate statistical significance and asterisks (*) indicate marginal significance.

a Parent (genetic and foster) and individual IDs were included as random effects in model.

b Only includes individuals that lived until Day 30 (metabolism measurement).

## 4 Discussion

Here, we manipulated the developmental environment of zebra finches to disentangle the impact of inheritance, incubation temperature, and post-hatch rearing conditions (i.e., cross-fostering) on the energy metabolism of embryos and offspring at nutritional independence (i.e., Day 30). We found that embryonic heart rate, a proxy of embryonic metabolism, was positively related to genetic father RMR and that embryos incubated at the higher incubation temperature had faster heart rates than those incubated at the lower temperature. Further, we found evidence that post-hatch offspring RMR is heritable and has a positive correlation with genetic mother RMR, although we found no relationship between offspring RMR and either foster parent RMR or incubation temperature. Lastly, we found that the metabolic cost of thermoregulation (i.e., TMR and ΔMR) had a lower heritability than RMR, but was positively related to genetic father TMR and ΔMR. Interestingly, foster mother TMR and ΔMR were negatively correlated with offspring TMR and ΔMR, respectively, but this relationship was only apparent when offspring were incubated at the lower temperature. This suggests that there are combined effects of the pre-natal environment and post-natal parental care on the metabolic cost of thermoregulation.

### 4.1 Effects of Incubation Temperature

As predicted, eggs that were incubated at the lower temperature had slower embryonic heart rates than those incubated at the higher temperature. Because embryonic heart rate should be an indicator of embryonic metabolism (Du et al., 2010; Sheldon et al., 2018), this suggests that low incubation temperatures lead to a lower embryonic metabolic rate. Our results agree with two other studies that have investigated the relationship between incubation temperature and embryonic heart rate (Rubin, 2019; Stier et al., 2020), and with one study that found that wood duck (*Aix sponsa*) eggs incubated at lower temperatures had lower daily embryonic oxygen consumption compared to those incubation at higher temperatures (DuRant et al., 2011). This lower energy expenditure during embryonic development should be related to slower developmental rates (Vedder et al., 2017; Sheldon and Griffith, 2018). Indeed, we found that eggs incubated at the lower temperature had a longer developmental duration (i.e., incubation period) than those incubated at the higher temperature (Table 1). Importantly, we still found a difference in embryonic heart rate between incubation temperatures when we corrected for differences in eggshell temperature and differences in developmental rate (i.e., developmental age vs. calendar age; see Supplementary Material). This suggests that incubation temperature alters physiology during development, more so than just a linear relationship with current temperature or developmental rate.

We predicted that offspring incubated at the low temperature would have greater post-hatch metabolic rates than those incubated at the control temperature because previous studies have found this effect of incubation temperature on both RMR (Nord and Nilsson, 2011; Wada et al., 2015) and TMR (DuRant et al., 2012) in birds. However, contrary to our predictions, offspring metabolic rate (RMR and TMR) was not affected by our incubation temperature treatments (control: 37.5°C; low: 36.3°C). This disagrees with some other avian studies. For example, Nord and Nilsson (2011) found that 14-day-old blue tits (*Cyanistes caeruleus*) incubated at a low temperature had higher RMR than those incubated at a warmer temperature and DuRant et al. (2012) found that 1-day-old wood ducks (*Aix sponsa*) incubated at a lower temperature had higher TMR than those incubated at a warmer temperature. To date, no study has investigated the effect of incubation temperature on zebra finch TMR, and two studies have examined the effects of incubation temperature on zebra finch RMR, with conflicting results. Supporting our findings, Berntsen and Bech (2021) found no difference in RMR among zebra finches that were incubated at 35.9°C and 37.9°C, measured at 15 and 45 days-old. In contrast, Wada et al. (2015) found that 25-day-old zebra finches incubated at 36.2°C had a higher RMR than those incubated at 37.4°C, although this effect was only found in females. In our study, we did not find an effect of sex on metabolic rate. In light of these conflicting results among species, it is possible that the effect of incubation temperature on metabolic rate is species-specific. For example, zebra finches may be a species that is more resistant to small changes in the embryonic thermal environment, and offspring develop similar physiological traits regardless of their developmental conditions. It is also possible that metabolic rate is more influenced by inheritance than by either the early developmental environment or sex, which could explain differences among zebra finch studies. Indeed, we found evidence that both RMR and TMR are heritable (see below). If our breeding parents displayed more genetic variation than those in the study of Wada et al. (2015), this could have masked any effects of incubation temperature and could explain the difference in results of our two studies. It should also be noted that there was relatively low hatching success in our study, although it was within the range of hatching success found in other captive zebra finch studies (e.g., von Engelhardt et al., 2004; Von Engelhardt et al., 2006; Criscuolo et al., 2011; Winter et al., 2013). Nevertheless, we cannot exclude the possibility that there was a selective process during hatching, and that all offspring that succeeded to hatch had similar metabolic rates, regardless of incubation temperature.

### 4.2 Relationship With Genetic Parents

Embryonic heart rate was positively related to genetic father RMR, suggesting that there could be a genetic component to embryonic metabolism. Although there is evidence that embryonic heart rate and metabolism vary among different genetic lines in poultry (Druyan, 2010), and that there are significant among-clutch differences in embryonic heart rate in wild zebra finches (Sheldon et al., 2018), this is the first study to our knowledge that has explicitly investigated the relationship between parental and embryonic metabolism in birds. Although the correlation between genetic parent metabolism and embryonic heart rate could also be due to maternal effects during egg formation, such as egg yolk composition (Ho et al., 2011), the relationship between embryonic heart rate and genetic mother RMR was not significant. In contrast, because the relationship with genetic father RMR was statistically significant, this suggests that maternal effects may not be as important as genetics for determining embryonic metabolism in zebra finches.

As predicted, we found evidence that offspring RMR was highly heritable (*h* ^*2*^ = 0.53). This agrees with other studies that show that RMR is a heritable trait. For example, both Rønning et al. (2007) and Nilsson et al. (2009) measured RMR heritability by using restricted maximum likelihood to compare RMR among siblings and found evidence that RMR was heritable in zebra finches (*h* ^*2*^ = 0.25) and blue tits (*h* ^*2*^ = 0.59), respectively. Further, using methods similar to that of our study (i.e., parent-offspring regression), Bushuev et al. (2011) found evidence for heritability of RMR (*h* ^*2*^ = 0.43) in pied flycatchers (*Ficedula hypoleuca*). Further in line with our results, Bushuev et al. (2011) found that offspring RMR was correlated with genetic parent RMR, but not foster parent RMR. The relationship between genetic parent and offspring RMR that we found in this study could also be due to non-genetic maternal effects, such as hormone deposition to the egg. Indeed, in contrast to what we found for embryonic heart rate, when we examined the RMR of the genetic mother and genetic father as separate factors, we only found a relationship between offspring RMR and genetic mother RMR, and not genetic father RMR. This suggests that pre-incubation maternal effects may play a large role in determining post-hatch offspring RMR. For example, zebra finch eggs with higher testosterone concentrations produce nestlings and adults with higher RMR (Tobler et al., 2007; Nilsson et al., 2011). If the mothers with higher RMR in our study also deposited more testosterone into their eggs, this could partly explain the relationship that we found between parent and offspring RMR.

In comparison to RMR, the metabolic cost of thermoregulation (i.e., TMR and ΔMR) was less heritable. It may be expected that TMR would have a lower heritability than RMR because it may be more variable due to its dependence on the insulation capacity of plumage. For example, the body feathers of juvenile birds have different structural properties than those of adult birds (Butler et al., 2008), which could mask relationships between the TMR of parents and young offspring. However, although the *h* ^*2*^ of TMR (*h* ^*2*^ = 0.38) and ΔMR (*h* ^*2*^ = 0.18) were not statistically significant, the *h* ^*2*^ of TMR was still within the range of those found for RMR (see above). Further, offspring TMR tended to be positively related to genetic father TMR, and offspring ΔMR was positively related to genetic father ΔMR. This is the first evidence that we are aware of for the heritability of TMR or ΔMR, and suggests that the metabolic expenditure associated with thermoregulation could be shaped by natural selection. However, in contrast to RMR, we found little evidence for non-genetic maternal effects because, when genetic mother and genetic father were tested as separate factors, genetic mother TMR and ΔMR were not related to that of their offspring. Thus, any non-genetic maternal effect that may have influenced RMR either did not translate into differences in thermoregulatory capacity, or was masked by other driving factors (e.g., post-hatch environment; see below).

### 4.3 Relationship With Foster Parents

Although there were no relationships between foster parent and offspring RMR, we found that foster mother TMR and ΔMR were negatively related to that of their foster offspring, but only for offspring in the low treatment. This suggests that the impact of foster mother metabolism, and thus post-hatch parental care, is not on RMR, but rather on the ability of offspring to increase their metabolic rate when faced with a thermal energetic challenge. Thus, the ability of parents to increase their metabolic rate in response to an energetic challenge may be important for effective post-hatch parental care. Because most studies focus on RMR, our results call for future studies to focus more on TMR.

Specifically, we found that the more energy that foster mothers expended on thermoregulation, the less energy that their foster offspring expended on thermoregulation. It is possible that this relationship can be explained by differences in nestling provisioning. For example, foster mothers with a high metabolic rate should also have a high investment in parental care (Daan et al., 1990; Koteja, 2004; Sadowska et al., 2013), and provide a better developmental environment for their offspring (e.g., more food provisioning; Nilsson, 2002). In zebra finches, a greater food supply during nestling development, as opposed to food restriction, is related to lower offspring metabolic rate later in life (Criscuolo et al., 2008; Careau et al., 2014). Thus, this could explain the negative relationship that we found between foster mother and offspring TMR and ΔMR. In our study, offspring growth rate between Day 0 and Day 30 was not correlated with foster mother TMR (*p* = 0.9), and thus we did not find evidence to support the hypothesis that foster mother TMR is positively related to food provisioning and/or better parental care. However, we did not measure parental care behavior (i.e., provisioning rates) in this study, and thus future studies are needed to determine whether there is a relationship between parental TMR and nestling provisioning rates, along with offspring growth rates and metabolism.

It is important to note that the relationships between foster mother and offspring TMR and ΔMR were only present in offspring incubated at the low temperature, and not the control temperature. This suggests that the impact of post-hatch care (e.g., nestling provisioning) is dependent on the quality of pre-natal care (i.e., incubation temperature). It is possible that offspring incubated at the control temperature are more resistant to differences in their post-hatch environment than those incubated at the low temperature, although little is known about how incubation temperature may influence trait plasticity or canalization. Future research is needed to investigate how different thermal environments shape avian thermoregulatory ability across generations, especially in the context of acclimation and adaptation in response to climate change (Nord and Giroud, 2020).

### 4.4 Relationship Between Heart Rate, Hatch Success, and Metabolic Rate Within Individuals

Embryonic heart rate is important because it can be used as a proxy for embryonic metabolic rate (Du et al., 2010), and can also provide insights into developmental rate, hatchling phenotype, and the effects of environmental stressors (reviewed in Sheldon et al., 2018)*.* Contrary to what we expected, we did not find that embryonic heart rate was related to offspring RMR at Day 30. However, to our knowledge, this is the first study that has investigated whether there is a relationship between embryonic heart rate and offspring metabolism later in life. Our results suggest that individual metabolic rate can change throughout different stages of development, and that embryonic heart rate cannot be used to predict later-life metabolic rate in zebra finches. Indeed, studies that have found a positive relationship between heart rate and metabolic rate measured these two traits at the same developmental stage (i.e., embryonic; Du et al., 2010; Ide et al., 2017; Goodchild et al., 2020), and one study did not find a relationship between heart rate and metabolism even when measured at the same developmental stage (i.e., embryonic and hatching; Sartori et al., 2017). Similarly, one study on zebra finches found no relationship between embryonic heart rate and post-hatch growth rate or activity levels (Sheldon and Griffith, 2018). Thus, it appears that embryonic heart rate may not be able to be extrapolated to phenotypic differences later in life.

However, when we investigated an endpoint closer to embryonic development—hatch success—we did find a relationship with embryonic heart rate. Eggs that hatched had greater embryonic heart rates than those that did not hatch, suggesting that heart rate may be an indicator of embryo quality or hatching probability. Although heart rate has been used in other studies to predict hatching date or to confirm embryonic mortality (reviewed in Sheldon et al., 2018), this is the first study to our knowledge that has explicitly linked the magnitude of embryonic heart rate to hatching probability. Because all individuals that hatch also have high heart rates as embryos, this could create a selective process for a particular metabolic functioning. It is possible that this could mask potential effects of the incubation treatment or parental care on offspring metabolic rate, and could also explain why we did not find some of the relationships that we had predicted (e.g., effect of incubation temperature on RMR, relationship with foster parent RMR).

## 5 Conclusion

In this study, we show that avian metabolic rate throughout development, from the embryo to nutritional independence, is related to parental inheritance, the pre-hatch environment (i.e., incubation temperature), and post-hatch conditions (i.e., foster parent). Revealing how these different factors are related to RMR and TMR sheds light on how metabolism and the energetic cost of thermoregulation can be shaped by environmental changes, parental care decisions, and natural selection. Although most studies to date focus on RMR, our study reveals important relationships with TMR, which could be particularly important in the context of climate change for understanding how the early thermal environment and parental care affect thermoregulatory ability, and the possibility that thermoregulatory ability can be shaped by natural selection. More work is needed to determine if the differences in RMR and TMR that we found in this study have effects on short- or long-term offspring fitness.

## Data Availability

The raw data supporting the conclusion of this article will be made available by the authors, without undue reservation.
