# Supplementary material for: Nature vs. Nurture: Disentangling the Influence of Inheritance, Incubation Temperature, and Post-Natal Care on Offspring Heart Rate and Metabolism in Zebra Finches
Source: Front Physiol. 2022 May 10;13:892154. doi: 10.3389/fphys.2022.892154 (PMC9127084; doi:10.3389/fphys.2022.892154)
Supplement: Supplementary file 1 [file DataSheet1.docx]

Supplementary Material

Appendix 1: Embryonic heart rate verification

**Purpose:** To verify that embryonic heart rate results are not driven by differences in eggshell temperature, embryo age, or embryo developmental stage.

# Methods

Because temperature is related to heart rate (Sheldon et al., 2018), we conducted a test where we measured egg surface temperature to correct embryonic heart rate measurements for differences in temperature. We incubated non-experimental eggs at a constant temperature of either 37.5°C (‘Control’: *N* = 13 eggs) or 36.3°C (‘Low’: *N* = 14 eggs). We measured egg surface temperature of eggs after 11, 12, and 13 days of incubation for ‘control’ eggs and after 12, 13, and 14 days of incubation for ‘low’ eggs. We chose to measure eggs one day apart from each other because control eggs develop faster than low eggs (i.e., shorter incubation period by one day), and thus embryonic development should be similar between the two incubation temperature treatments if measured one day apart. At each time point, we measured egg temperature using an infrared camera (Fluke TiX580) after 30, 60, 90, 120, 150, and 180 seconds of removing the egg from the incubator. We also noted whether the egg was alive, infertile, or dead (i.e., partly developed, but no detectable heart rate).

## Statistical analyses

To determine after how many seconds after being removed from the incubator that eggs incubated at 37.5°C (‘control) and 36.3°C (‘low’) reached the same surface temperature, we built a linear mixed effects model with egg surface temperature as the dependent variable, incubation temperature, egg age (‘reading 1, 2, or 3’), egg mass, and the time to take the measurement (seconds) as independent variables, and genetic parent ID and individual ID as random effects. We also included whether the egg was infertile (yes or no) or dead (yes or no) in the model at first, and we found that infertile eggs significantly differed in temperature compared to fertile eggs (*p* = 0.014), but dead eggs did not differ from eggs that were alive (*p* = 0.15). Thus, we excluded infertile eggs (control: 4 infertile eggs; low: 1 infertile egg) from this analysis.

After determining after how many seconds ‘control’ and ‘low’ eggs had the same surface temperature (see *Results*), we then reanalyzed our embryonic heart rate data, using the same model as described in the main paper, but only including data from the specified time points (i.e., 50 – 70 sec for ‘low’ eggs and 110 – 130 sec for ‘control’ eggs).

Further, although in the main manuscript we chose to measure heart rate when eggs were the same ‘developmental age’ (i.e., considering the one-day difference in development time between control and low eggs), we also verified that this decision did not change our results. Thus, we reran models while only including eggs that were the same ‘calendar age’ (i.e., we only included measurements on Days 12 and 13, which we had for every egg regardless of its incubation temperature).

# Results

We found that eggshell temperatures decreased as time passed after eggs were taken out of the incubator (*p* < 0.001; Table S1), decreased as eggs aged (*p* < 0.001; Table S1), increased as egg mass increased (*p* < 0.001), and that eggs incubated at the low temperature had colder eggshell temperatures than those incubated at the control temperature (*p* = 0.0029; Table S1). Importantly, *post-hoc* pair-wise comparisons (*emmeans*) revealed that eggshells from the control group after 120 sec out of the incubator reached the same temperature as eggshells from the low group after 60 sec (*p* = 1.0; Fig. S1).

To correct for differences in eggshell temperature, we included heart rate measurements that were taken after 110-130 seconds for eggs from the control temperature, and after 50-70 seconds for the low temperature. Using this subset of data, we found that our results were similar to those of the complete data set (Table S2; Fig. S2). Further, when we reanalyzed our data using ‘calendar age’ instead of ‘developmental age’, our results also remained consistent (Table S2; Fig. S2). We report all combinations of our analysis using the full dataset, the dataset corrected for eggshell temperature, developmental age, and calendar age in Table S2 and Fig. S2, and find that our results remain consistent.

# Supplementary Tables

**Table S1**. Linear mixed effects model investigating the relationship between eggshell temperature (°C) and the amount of time that the egg spent out of the incubator (sec).

|  | *Full model^a^* | |
| --- | --- | --- |
|  | *N_control_* = 13 eggs; 230 readings  *N_low_* = 14 eggs; 246 readings | |
| **Term** | ***F*** | ***p*** |
| Incubation temperature | 6.19 | **0.020** |
| Egg age* | 11.70 | **<0.001** |
| Time to measurement | 137.21 | **<0.001** |
| Egg mass | 9.60 | **0.0051** |
| Infertile | 6.81 | **0.016** |
| Dead | 0.35 | 0.56 |
|  | *Reduced model^b^* | |
|  | *N_control_* = 9 eggs; 162 readings  *N_low_* = 13 eggs; 228 readings | |
|  | ***F*** | ***p*** |
| Incubation temperature | 11.71 | **0.0029** |
| Egg age^c^ | 28.24 | **<0.001** |
| Time to measurement | 151.46 | **<0.001** |
| Egg mass | 15.97 | **<0.001** |

Bold values indicate statistical significance and asterisks (*) indicate marginal significance

^a^Linear mixed effects model with egg ID as the random effect

^b^Infertile eggs excluded; dead eggs included in analysis but factor ‘dead’ dropped from final model because not significant

^c^Egg age was classified as ‘reading 1, 2, or 3’, since heart rate was measured at different ages depending on the incubation temperature

**Table S2.** Linear mixed effects models investigating the relationship of embryonic heart rate with incubation temperature using different data subsets

|  | **Calendar ages; all data** | | **Calendar ages; corrected for temperature** | | **Developmental ages; all data^a^** | | **Developmental ages; corrected for temperature** | |
| --- | --- | --- | --- | --- | --- | --- | --- | --- |
|  | *Full models^b^* | | | | | | | |
|  | *N_control_* = 103 eggs; 527 readings  *N_low_* = 117 eggs; 677 readings | | *N_control_* = 20 eggs; 25 readings  *N_low_* = 95 eggs; 148 readings | | *N_control_* = 107 eggs; 839 readings  *N_low_* = 117 eggs; 997 readings | | *N_control_* = 41 eggs; 53 readings  *N_low_* = 109 eggs; 230 readings | |
| **Term** | ***F*** | ***p*** | ***F*** | ***p*** | ***F*** | ***p*** | ***F*** | ***p*** |
| Incubation temperature | 134.11 | **<0.001** | 25.16 | **<0.001** | 175.97 | **<0.001** | 3.67 | 0.057* |
| Egg age | 41.01 | **<0.001** | 21.80 | **<0.001** | 20.31 | **<0.001** | 20.36 | **<0.001** |
| Time to measurement | 696.06 | **<0.001** | - | **-** | 915.67 | **<0.001** | - | - |
| Egg mass | 2.85 | 0.094* | 5.27 | **0.025** | 5.35 | **0.023** | 6.34 | **0.014** |
| Inc temp x Egg age | 1.05 | 0.30 | 8.68 | **0.004** | 10.84 | **<0.001** | 7.10 | **0.001** |

Bold values indicate statistical significance and asterisks (*) indicate marginal significance

^a^Same dataset from the main paper

^b^Parent ID and Individual ID were included as random effects in all models

# Supplementary Figures

**Figure S1.** Relationship between time spent out of the incubator (sec) and eggshell temperature (°C; mean ± SE). Eggs incubated at the control temperature (37.5°C) are represented in black and those incubated at the low temperature (36.3°C) in gray.

**Figure S2.** Relationships between embryonic heart rate and incubation temperature using different data subsets. Eggs incubated at the control temperature (37.5°C) are represented in black and those incubated at the low temperature (36.3°C) in gray. Panel A uses all data and the calendar age of eggs (i.e., number of days in incubator). Panel B uses the subset of data where all eggs have the same eggshell temperatures (see text and Fig. S1) and the calendar age of eggs. Panel C uses all data and the developmental age of eggs (i.e., low eggs were measured one day later to correct for their slower developmental rate; see text). Note that Panel C is the same as Figure 1 in the main manuscript. Panel D uses the subset of data where all eggs have the same eggshell temperatures and developmental ages. For developmental ages, eggs from the control treatment were measured on Days 11, 12, and 13, and those from the low treatment were measured on Days 12, 13, and 14.
